# Left Paraduodenal Hernia: A Rare Complication following Laparoscopic Appendectomy

**DOI:** 10.1155/2017/3913784

**Published:** 2017-12-06

**Authors:** Mathew A. Kozman, Oliver M. Fisher

**Affiliations:** ^1^Department of General Surgery, St George Hospital, Kogarah, NSW, Australia; ^2^School of Medicine, University of Notre Dame, Sydney, NSW, Australia

## Abstract

Paraduodenal hernias are rare congenital internal hernias accounting for <2% of intestinal obstruction. Left paraduodenal hernias (LPDHs) into the fossa of Landzert are the more common type and result from abnormal rotation of the midgut and failure of peritoneal fusion. Sequelae of these hernias usually occur spontaneously in the 4th or 5th decade of life and are more common in males and have a significant risk of incarceration and subsequent strangulation. We describe a case of a 15-year-old female who develops a LPDH following laparoscopic appendectomy, resulting in jejunal incarceration and subsequent small intestinal obstruction. The patient discussed is from an atypical demographic, being young and female. In addition, the precipitating event prompting incarceration of the hernia appears to be the application of pneumoperitoneum, placement in the Trendelenburg position, and manipulation of small intestine for the purpose of facilitating laparoscopic appendectomy. To our knowledge, this is the first reported case of LPDH exacerbated by laparoscopic procedure.

## 1. Introduction

Paraduodenal hernias are rare congenital internal hernias accounting for <2% of intestinal obstruction, most commonly involving jejunum [1]. Left paraduodenal hernias (LPDHs) into the fossa of Landzert are the more common type and result from abnormal rotation of the midgut and failure of peritoneal fusion [2, 3]. Sequelae of these hernias usually occur spontaneously in the 4th or 5th decade of life and are more common in males [2, 4]. Patients with LPDH have a significant risk of incarceration and subsequent strangulation, associated with high mortality rates [5]. We report on an atypical event of incarcerated LPDH.

## 2. Case Report

A 15-year-old female presented with a 12-hour history of gradual onset right iliac fossa abdominal pain associated with nausea, vomiting, anorexia, and fevers. She denied urinary symptoms. Her last menstrual period was 3 months prior and was an irregular occurrence. She had no similar previous episodes. She had no significant past medical or surgical history and took no regular medications.

On examination, she was alert, orientated, and appeared well. Vital signs were within normal limits, and she was afebrile. Abdominal examination revealed tenderness in the lower abdomen, worse in the right iliac fossa. Rovsing's sign was present.

Investigations revealed a normal urine analysis, and subsequent culture was negative. White blood cell count was 19.1 × 109/L with neutrophilia. C-reactive protein was 3.0 mg/L. Abdominal ultrasound showed a noncompressible, avascular, hypoechoic tubular structure with well-defined margins measuring 9.2 mm, extending from the mid-pelvis to the left side. This was suspicious for appendicitis.

The patient underwent a diagnostic laparoscopy and appendectomy. This was performed using a 10 mm infraumbilical hasson port and two further 5 mm working ports. The patient was positioned supine with bed tilted head-down (Trendelenburg position) and rolled leftward. High-flow carbon dioxide insufflation of 12 mmHg was used, and small intestine was manoeuvred out of the pelvis into the upper abdomen. Early acute appendicitis with no perforation was found, and laparoscopic appendectomy was performed. Histopathology confirmed acute appendicitis.

The following day, the patient complained of severe and worsening abdominal pain. She was unable to tolerate oral intake. On examination, she appeared to be in pain. Tachycardia of 120 beats per minute was noted; otherwise, vital signs were within normal limits. Her abdomen was distended and tender with signs of generalised peritonism. Blood tests were unremarkable, with reduced white cell count compared with initial presentation. Due to this clinical picture, no further imaging studies were arranged, and she was taken back to the operating theatre for exploratory laparoscopy.

**Figure 1:**
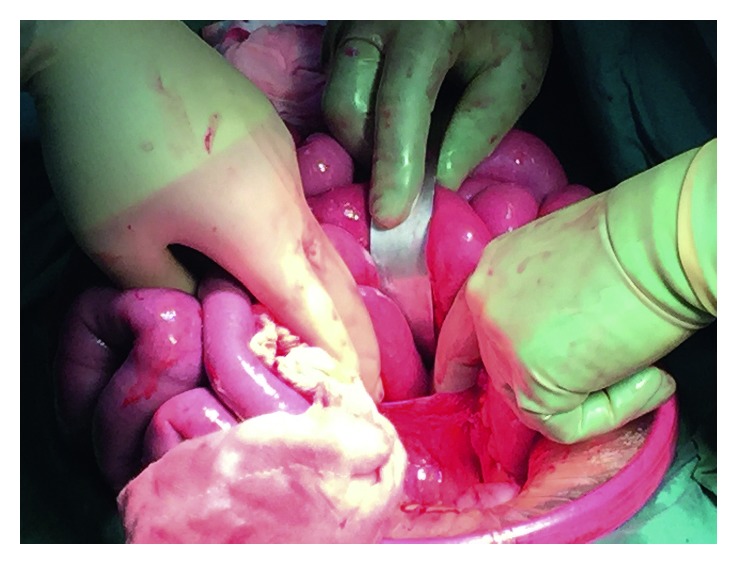
Intraoperative photograph showing left paraduodenal hernia: left paraduodenal hernia (LPDH) occurring when small intestine prolapses posteroinferiorly into this fossa (of Landzert), which is bounded by the fourth part of the duodenum, the posterior peritoneum, the inferior mesenteric vein, and left branches of the middle colic artery. LPDH being retracted in order to facilitate reduction of the hernial contents, namely, small intestine.

**Figure 2:**
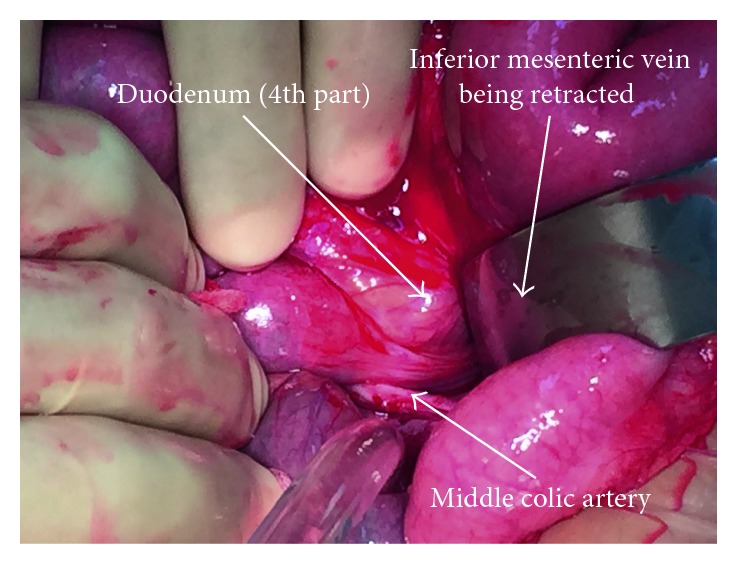
Intraoperative photograph showing left paraduodenal hernia: left paraduodenal hernia (LPDH) occurring when small intestine prolapses posteroinferiorly into this fossa (of Landzert), which is bounded by the fourth part of the duodenum, the posterior peritoneum, the inferior mesenteric vein, and left branches of the middle colic artery. LPDH borders displayed.

Upon laparoscopy, dilated small intestinal loops and copious free fluid were found. Conversion to laparotomy was performed due to impeded laparoscopic view. This revealed a small bowel obstruction with transition point at the paraduodenal region. An LPDH diagnosed with a long segment of jejunum was seen herniating through a defect to the left of the fourth part of the duodenum and accumulating in the fossa of Landzert (Figures 1 and 2). Reduction of the hernia was performed, ensuring preservation of the inferior mesenteric vein and middle colic artery branches. No widening of the hernia neck was required to successfully reduce the hernia. An approximately 15 cm segment of jejunum displayed signs of ischaemia but was viable upon hernia reduction and warm pack wrapping. The peritoneal hernia sac was excised, and the paraduodenal hernia orifice was closed using 2-0 vicryl and the sac as a patch. The patient made an uneventful recovery and was discharged home 5 days later.

## 3. Discussion

Internal hernias result from protrusion of the viscera through an opening in the peritoneum or mesentery [6]. They account for <2% of intestinal obstruction [1]. Paraduodenal hernias account for 30–50% of all internal hernias, which are also the most common type of congenital internal hernias [5]. Paraduodenal hernias into the left paraduodenal fossa of Landzert are three times more common than those into the right paraduodenal fossa of Waldayer [2]. The left paraduodenal fossa results from failure of mesenteric fusion with the parietal peritoneum and malrotation of the midgut resulting in development of a potential space [3]. LPDH occurs when small intestine prolapses posteroinferiorly into this fossa (of Landzert), which is bounded by the fourth part of the duodenum, the posterior peritoneum, the inferior mesenteric vein, and left branches of the middle colic artery [7, 8]. This may result in small bowel incarceration, obstruction, and subsequent ischaemia.

Patients most commonly present in the 4th to 5th decades or life [2], and there is a 3 : 1 male preponderance [4]. Presentation is variable depending on the severity of the hernia sequelae and the presence of obstruction [2]. Approximately 50% of patients recall previous recurring abdominal pain of nonspecific nature [9]. As such, the entity poses a diagnostic challenge, with majority of cases identified only at operation [10]. Patients with LPDH have a 50% lifetime risk of hernia incarceration with 20–50% mortality for acute presentations; hence, operative management is recommended regardless of symptoms [5].

The most effective preoperative diagnostic tool is the computed tomography (CT) scan [11]. This may reveal a cluster of small bowels at the ligament of Treitz with or without associated findings of small intestine obstruction [2]. An additional compression on the posterior stomach and distal duodenum results in inferior displacement of transverse colon and shifting of the mesenteric truck to the right [12]. However, instances of acute abdomen warrant omission of preoperative imaging and immediate exploratory laparotomy.

Treatment methods reported in the literature include laparoscopy or laparotomy and repair. However, exploratory laparotomy is more often reported, especially in the setting of an acute complication such as strangulation, perforation, or large distension from obstruction [13]. In addition, laparotomy may be more appropriate in circumstances where laparoscopy may not be possible or dangerous such as significant adhesions, haemodynamic instability, and contraindication to pneumoperitoneum [13]. Nonetheless, the laparoscopic approach to management of this condition has become increasingly prevalent in the literature [14]. Regardless of the approach, basic principles of hernia repair are adopted, namely, reduction of hernia contents and repair of hernia defect [9]. Excision of the hernia sac has been described but is not mandatory given the potential for injury to the colic vessels [14]. Correct identification and preservation of the vascular structures that constitute the hernia neck is essential [12]. Rarely, widening of the hernia neck is required to reduce the contents and may involve incision of constricting peritoneal fold inferiorly and even division of the inferior mesenteric vein in more difficult cases [15].

A literature search of PubMed back to 1980 discovered only approximately 50 reported cases of intestinal obstruction secondary to a LPDH. However, to our knowledge, this is the first reported case of LPDH exacerbated by laparoscopic pneumoperitoneum and intraoperative patient positioning. This case is particularly interesting for a few reasons. Firstly, the patient demographic is not typical of this disease. Our patient was a young female, while reported cases more commonly describe males in the 4th to 5th decades of life. Secondly, the patient described no symptoms at any time prior to the day of her appendectomy. Although the patient clearly possessed the congenital anomaly of a left paraduodenal fossa, the application of pneumoperitoneum, placement in the Trendelenburg position, and manipulation of small intestine for the purpose of facilitating laparoscopic appendectomy appear to have precipitated the LPDH and subsequent incarceration. In this instance, the diagnosis was not made prior to laparotomy as in most cases presenting with an acute abdomen. Although diagnostic laparoscopy was performed upon return to theatre, gross small bowel distension from obstruction prevented further progression via the laparoscopic approach.
